# A Low-Testosterone State Associated with Endometrioma Leads to the Apoptosis of Granulosa Cells

**DOI:** 10.1371/journal.pone.0115618

**Published:** 2014-12-23

**Authors:** Yoshihiro J. Ono, Akiko Tanabe, Yoko Nakamura, Hikaru Yamamoto, Atsushi Hayashi, Tomohito Tanaka, Hiroshi Sasaki, Masami Hayashi, Yoshito Terai, Masahide Ohmichi

**Affiliations:** Department of Obstetrics and Gynecology, Osaka Medical College, Osaka, Japan; University of Nebraska Medical Center, United States of America

## Abstract

Although endometriosis is suspected to be a cause of premature ovarian insufficiency (POI), the mechanism(s) underlying this process have not been elucidated. Recently, androgens were shown to promote oocyte maturation and to play a role in folliculogenesis. In addition, several reports have documented low testosterone levels in the follicular fluid obtained from endometriosis patients. We therefore examined whether the low levels of serum testosterone are associated with the apoptosis of granulosa cells in follicles obtained from endometriosis patients. Serum samples were collected from 46 patients with endometriosis and from 62 patients without endometriosis who received assisted reproductive therapy. Specimens of the ovaries obtained from 10 patients with endometrioma were collected using laparoscopy. The mean serum testosterone concentration in the patients with endometriosis was significantly lower than that observed in the patients without endometriosis. Furthermore, high expression of a pro-apoptotic Bcl-2 member, BimEL, in the follicles was found to be associated with a low serum testosterone level. We clarified the underlying mechanisms using a basic approach employing human immortalized granulosa cells derived from a primary human granulosa cell tumor, the COV434 cell line. The *in vitro* examination demonstrated that testosterone inhibited apoptosis induced by sex steroids depletion via the PI3K/Akt-FoxO3a pathway in the COV434 cells. In conclusion, we elucidated the mechanism underlying the anti-apoptotic effects of testosterone on granulosa cells, and found that a low-testosterone status is a potentially important step in the development of premature ovarian insufficiency in patients with endometriosis.

## Introduction

Endometriosis is a chronic benign disease characterized by the presence of endometrium-like tissue outside the uterine cavity, primarily on the ovaries. It is a major cause of symptoms, such as pelvic pain, dysmenorrhea, dyspareunia and infertility, affecting 6–10% of females of reproductive age and at least one-third of those with infertility and often relapsing after surgery [1]–[3]. The aim of most medical treatments for endometriosis is to alleviate pain and other symptoms, reduce the size of the endometriotic lesions and improve the patient’s quality of life without causing problems with infertility; however, there are limited therapeutic options for infertile patients. Although endometriosis is generally thought to be related to infertility, its actual impact on fecundity and the mechanisms underlying this effect are less clear. Several controlled trials have reported rates of reduced fecundity among patients with endometriosis of 2–10% [4]. Furthermore, experience with IVF has demonstrated that poor pregnancy outcomes in endometriosis patients are associated with a poor ovarian reserve, reduced oocyte retrieval, lower oocyte and embryo quality and impaired implantation with decreased endometrial receptivity, particularly in those with advanced-stage disease [5], [6]. Although endometriosis is suspected to be a cause of premature ovarian insufficiency (POI), no effective treatment has been established to prevent POI in individuals with endometriosis [7].

For decades, androgens have been believed to play a negative or dispensable role in meiotic maturation in mammals [8], [9]. However, these hormones were recently shown to promote oocyte maturation in mice [10]–[13]. In addition, the broad localization of androgen receptors (ARs) in ovarian cells [14], [15] suggests that androgens play a role in folliculogenesis [10]. In AR knockout mice, intensive granulosa apoptosis occurs during the periovulatory period [16], and these mice develop the POI phenotype with a loss of follicles [17]. Interestingly, several reports have also documented low testosterone levels in the follicular fluid obtained from endometriosis patients [18]. Furthermore, an abnormal level of cytochrome P450 aromatase (CYP19a1), which converts testosterone to estradiol, has been demonstrated in endometriotic implants, resulting in increased estradiol production [19]. Several independent studies have also revealed that CYP19a1 is overexpressed in both the eutopic and ectopic endometrium of patients with endometriosis [20]–[22]. These findings prompted us to hypothesize that a state of low testosterone in endometriosis patients promotes granulosa cell apoptosis resulting in a poor ovarian reserve.

In the present study, we therefore analyzed the serum testosterone levels in endometriosis patients, and evaluated whether a low serum testosterone level correlates with the apoptosis of granulosa cells. Furthermore, we assessed whether a favorable testosterone level prevents granulosa cell apoptosis and clarified the underlying mechanisms using a basic approach employing human immortalized granulosa cells derived from a primary human granulosa cell tumor, the COV434 cell line [23].

## Materials and Methods

### Patient and sample collection

We recruited a total of 118 patients treated between January 2011 and July 2013 for this study: 108 patients were evaluated for the hormone levels in their serum and follicular fluid during assisted reproductive therapy, and 10 patients were evaluated for a histological analysis of the ovarian samples after unilateral oophorectomy, as described later. All subjects were under treatment in the Department of Obstetrics and Gynecology of Osaka Medical College, and had not received prior treatment at other facilities. This study was a retrospective analysis of human tissue samples approved by the Institutional Review Board of Osaka Medical College, and written informed consent was obtained from all participating patients.

A total of 108 patients who received assisted reproductive therapy were included in the evaluation of differences in the serum hormone levels between those with and without ovarian endometriomas. Participants diagnosed with clinical endometriosis associated with ovarian endometriomas (n = 46) were analyzed as the endometriosis group, while those with male factor infertility (n = 35) or tubal factor infertility (n = 27) were analyzed as the control group. In the endometriosis group, transvaginal ultrasonography was performed in all patients, and the presence of ovarian endometrioma was confirmed on magnetic resonance imaging (MRI) based on the detection of high-intensity areas on both T1- and T2-weighted images. Out of 46 patients in the endometriosis group, 31 patients had bilateral endometrioma and 15 patients had unilateral endometrioma. Blood samples were collected on days 2–3 of the spontaneous menstrual cycle.

In order to evaluate the role of the serum testosterone level in the apoptosis of granulosa cells in the endometriosis patients, 10 subjects with ovarian endometrioma who underwent unilateral salpingo-oophorectomy were also recruited for the analysis. All of the patients had rapid growth of an endometrioma with suspected malignancy. Therefore, unilateral salpingo-oophorectomy was performed, following by a pathological evaluation. Fortunately, malignancy was ruled out in all of the cases in this study. In these patients, blood samples were collected on days 2–3 of the spontaneous menstrual cycle prior to surgery, and simultaneous sampling of ovarian tissue was performed during laparoscopic surgery. The diagnosis of endometriosis was confirmed histologically.

The inclusion criteria were as follows: an age greater than 20 and no more than 40 years at the time of the treatment procedure; a regular menstrual cycle (with an interval of 24–35 days); a body mass index (BMI) between 20 and 30 kg/m^2^; both ovaries present; no history of ovarian surgery; no evidence of past or recent pelvic inflammatory disease; no evidence of endocrine disorders; and no history of hormone treatment for at least 12 months at baseline.

### Collection of follicular fluid

The 108 patients underwent a long GnRH agonist protocol with a luteal-phase start. Ovarian stiumulation with recombinant FSH was begun after pituitary suppression was confirmed. hCG was administered when there were at least two follicles of ≥20 mm average diameter. Transvaginal ultrasound-guided follicular aspiration was performed 36 hours after the hCG injection. Individual aspiration was used to collect oocytes, and each follicle was collected in a different tube. To avoid contamination from blood, flush medium, or mixed follicular fluid during oocyte retrieval, only the follicular fluid from the first retrieved follicle which contains a single oocyte-cumulus complex was collected. Therefore, one follicular sample per patients was used for analysis. Samples of follicular fluid were centrifuged at 2,000 g for 10 minutes and the surpernatants stored at −80°C for further analysis.

### Hormone measurements

The FSH, LH, estradiol (E2) and testosterone (Ts) concentrations were determined based on an electrochemiluminescence immunoassay (ECLIA) using Elecsys FSH II, LH, Estradiol II and Testosterone II kits, respectively, according to the manufacturer’s instructions. All assays were performed on a Cobas e601 immunoassay analyzer (Roche Diagnostics GmbH, Mannheim Germany). The limit of detection was 0.1 mIU/mL for FSH, 0.1 mIU/mL for LH, 5 pg/ml for E2 and 0.025 ng/dl for Ts. The intra- and interassay coefficients of variation (CVs) for estradiol and testosterone were both less than 10%. Cross-reactivity with other relevant metabolites was detected in less than 0.86% of the samples in the Estradiol II assay and 3.22% of the samples in the Testosterone II assay. The serum levels of CA125 were determined using a chemiluminescent enzyme immunoassay on the Architect Analyzer (Abbott laboratories, Chicago, IL), which has an inter-assay precision for CA125 of 2.85% (35.6 mU/mL). We have considered 35 U/mL as the upper limit of normal for CA 125.

### Materials

Testosterone and flutamide (an androgen receptor inhibitor) were purchased from Wako Pure Chemical Industries (Osaka, Japan). Aromatase Inhibitor I was purchased from Merck Millipore (Billerica, MA, USA). LY 294002 was purchased from Sigma-Aldrich (St. Louis, MO, USA). All rabbit monoclonal anti-human Akt antibodies, monoclonal anti-human phospho-Akt antibodies, rabbit monoclonal anti-human FoxO3a antibodies, monoclonal anti-human phospho-FoxO3a antibodies, rabbit monoclonal anti-human beta actin antibodies and rabbit polyclonal anti-human Bim antibodies used for immunoblotting and immunohistochemistry were purchased from Cell Signaling Technology, Inc. (Danvers, MA, USA). Rabbit monoclonal anti-human FSH receptor antibodies were purchased from Abcam (Cambridge, MA, USA).

### Immunohistochemical analysis

The ovarian tissue samples obtained from the patients who underwent unilateral salpingo-oophorectomy for endometrioma (n = 10) were corrected for immunohistochemistry. All of the patients had rapid growth of an endometrioma with suspected malignancy; therefore, unilateral salpingo-oophorectomy was performed, followed by a pathological evaluation. The ovarian samples were fixed in formalin and embedded in paraffin. Some sections were stained with hematoxylin and eosin for a histological examination with light microscopy to confirm their benign nature. Deparaffinized and antigen retrieval were performed, then the sections were subsequently incubated at 4°C for 12 hours with anti-BimEL (C34C5) rabbit antibodies (1∶100 dilution; Cell signaling Technology) or anti-FSH receptor antibodies (1∶100 dilution; Abcam). Negative control sections were processed using non-specific IgG (A0423, Dako). The antigen-antibody complexes were identified using the Universal DAKO LSAB2-labeled streptavidin-biotin peroxidase kit (K0609 DAKO). The follicles were classified into two stages, as follows: 1) primordial, identified by the presence of an oocyte partially or completely encapsulated by flattened squamous cells; or 2) growing (primary and secondary), with single or multiple layers of cuboidal granulosa cells around the oocyte. Staining was detected using a Carl Zeiss (Gottingen, Germany) Axiophot microscope. Photographs were taken of six different areas, and were further processed using the Adobe Photoshop software program (Adobe Systems, Unterschliessheim, Germany). Granulosa cells of each follicle were scored negative if absolutely no staining was present compared to adjacent control tissue sections. If staining was present in only some granulosa cells, the follicle was also given a negative score. Follicles were scored positive if specific BimEL staining was present in almost all granulosa cells. The ratio of the number of follicles positively stained with BimEL to the total number of primordial and growing follicles was determined in six different fields in each specimen. At least total 100 follicles were counted in each patient. Toya et al. has shown that the mean rate of apoptosis in granulosa cells obtained from the patients with endometriosis was as high as 20% [24], using flow cytometric analysis. Therefore, the specimens with a BimEL-positive rate of more than 20% were classified as the BimEL-positive group, and the specimens with staining in less than 20% of the specimens were was classified as the BimEL-negative group.

### Cell culture

Human immortalized granulosa cells derived from a primary human granulosa cell tumor, the COV434 cell line [25], were purchased from the European Collection of Cell Cultures (Salisbury, UK). The COV434 cells were grown in Dulbecco’s modified Eagle’s medium (DMEM) supplemented with 10% fetal calf serum (FCS; Gibco, Basel, Switzerland) and penicillin/streptomycin (50 µg/ml) in an atmosphere of 5% CO_2_ at 37°C. Prior to the MTS assay, Western blotting analysis and RNA extraction, the COV434 cells were incubated in phenol red-free DMEM supplemented with 2% of Charcoal Stripped Fetal Bovine Serum (Equitech-Bio, Kerrville, TX) for 12 hours.

### RNA extraction, cDNA synthesis and RT-PCR

Total RNA was obtained cultured cells using the RNeasy Mini kit (Qiagen, Germantown, MD, USA), and 2 µg was subsequently reverse transcribed with Superscript II RNase H-reverse transcriptase (Invitrogen, Carlsbad, CA, USA) using random primers, according to the manufacturer’s instructions. cDNA was then amplified using Taq DNA polymerase (Roche Diagnostics, Mannheim, Germany). The primers were as follows: BimEL: 5′-GGCGTATCGGAGACGAGTTT -3′ (forward) and 5′-ACCATTCGTGGGTGGTCTTC-3′ (reverse), Beta-actin: 5′-AGC CAC ATC GCT CAG ACA-3′ (forward) and 5′-GCC CAA TAC AC CAA ATC C-3′ (reverse).

### Cell viability assay

The COV434 cells were seeded on 96-well plates at a density of 2×10^4^ cells per well in phenol red-free DMEM supplemented with 2% Charcoal Stripped Fetal Bovine Serum for 12 hours under steroid-free conditions. The cells were then incubated for 24 and 48 hours in the presence or absence of 10 or 100 nM testosterone with or without 5 µM of flutamide. The vehicles (ethanol or dimethyl sulfoxide) were used at a final concentration of 0.1% or less. CellTiter 96 AQueous (MTS) One Solution reagent (Promega, Tokyo, Japan) was added to each well, and the absorbance at 490 nm was recorded using a The Corona SH-1000Lab absorbance microplate reader (Corona Electric Co. Inc., Ibaraki, Japan). The number of cells was subsequently calculated using a standard curve correlating the absorbance to the cell count determined under a microscope. All experiments were performed in quadruplicate, and the cell viability was expressed as the ratio of the number of viable cells treated with testosterone stimulation to that of cells treated without stimulation.

### Detection of apoptotic cells

The apoptosis of granulosa cells was assessed under various conditions according to a caspase activity assay using the CspACETM FITC-VAD-FMK In Situ Marker (Promega, WI, USA), in accordance with the manufacturer’s instructions. The slides were analyzed with a confocal laser scanning microscope (Zeiss 410, Carl Zeiss GmbH, Gottingen Germany), and a flow cytometry analysis was conducted using FACScan in order to quantify the level of apoptosis based on the rate of testosterone depletion. Approximately 10,000-gated events were analyzed per sample. The results were analyzed using the Windows Multiple Document Interface flow cytometry applications software program, and the rate of apoptosis was calculated.

### Western blot analysis

COV434 cells were incubated in phenol red-free DMEM supplemented with 2% Charcoal Stripped Fetal Bovine Serum for 12 hours. The cells were then incubated for 24 hours in the presence or absence of 10 or 100 nM testosterone with or without 5 µM of flutamide. The cells were subsequently washed twice with ice-cold phosphate-buffered saline and lysed using Pierce RIPA Buffer (Thermo Fisher Scientific, MA, USA). Equal amounts of whole cell proteins were separated via SDS polyacrylamide gel electrophoresis and electrotransferred to nitrocellulose membranes. Western blot analyses were then performed with various specific primary antibodies; the immunoreactive bands in the immunoblots were visualized with horseradish peroxidase-coupled immunoglobulin using an enhanced chemiluminescence Western blotting system (ECL Plus, GE Healthcare Life Sciences, Pittsburgh, PA, USA).

### Statistics

All experiments were performed in triplicate, except for the proliferation assay. The statistical calculations were performed using the JMP statistical software package (SAS Institute, Cary, NC). An analysis of variance (ANOVA) was used for group comparisons, whereas comparisons of the peripheral blood samples were made using Wilcoxon’s test as a non-parametric test. The statistical significance of each difference was determined using the Kruskal-Wallis and Mann-Whitney U test or paired t-test, as appropriate. A *p* value of <0.05 was considered to be statistically significant.

## Results

### The serum levels of testosterone were significantly lower in the participants with endometriosis

The clinical characteristics of the participants are presented in Table 1. There were no relevant group differences in age, BMI, or the serum FSH, LH or estradiol concentrations at baseline; however, the serum testosterone concentrations were significantly lower in the endometriosis group (0.19±0.14 ng/ml) than in the control group (0.43±0.22 ng/ml, p = 0.0012) at baseline.

**Table 1:** Baseline patient characteristics.

	Control	Endometriosis	*p* value
Patient number	62	46	
Age (year)	35.6±3.2	35.2±3.7	NS
Body mass index (kg/m^2^)	20.3±3.2	20.6±2.8	NS
Serum FSH (mlU/ml)	8.0±4.4	10.4±3.9	NS
Serum LH (mlU/ml)	5.4±3.9	4.0±2.4	NS
Serum Estradiol (pg/ml)	50.7±21.9	35.7±20.8	NS
Serum Testosterone (ng/ml)	0.43±0.22	0.19±0.14	0.0012
Follicular fluid Testosterone (ng/ml)	17.04±4.94	9.16±3.96	0.0003

NS, not significant.

### A higher expression of BimEL in the follicles correlated with a state of low serum testosterone among the patients with endometriosis

**Figure 1:**
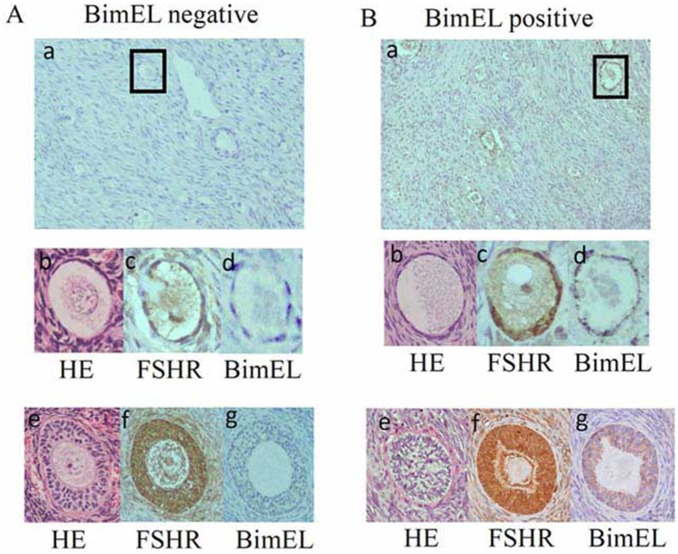
The detection of apoptosis using BimEL staining in primordial and growing follicles in endometriosis patients. Representative examples of sequential immunohistochemical sections stained with hematoxylin and eosin (b and e), anti-BimEL Abs (a, d, and g) and anti-FSH receptor Ab (c and f). The granulosa cells of each follicle were scored “negative” if there was absolutely no staining for BimEL, or if staining was present in only some granulosa cells. Follicles were scored “positive” if specific BimEL staining was present in almost all granulosa cells. The ratio of the number of follicles positively stained for BimEL to the total number of primordial and growing follicles was determined in six different fields in each specimen. At least total 100 follicles were counted in each patient. The specimens with a BimEL-positive rate higher than 20% was classified as the BimEL-positive group (panel B), and the specimens with staining of less than 20% of the specimen were classified as the BimEL-negative group (panel A).

In order to evaluate the effects of a low serum testosterone state at baseline on the follicles in the patients with endometriosis, ten endometriosis participants were newly recruited who underwent unilateral oophorectomy, and a histological analysis of the ovarian samples was performed. B-cell lymphoma 2 (Bcl-2) family proteins are considered to be major regulators of apoptosis [26], and one of the predominant isoform of pro-apoptotic Bcl-2 members is termed Bim extra-long (BimEL) [27]. According to data generated from different genetic models, the BimEL expression presumptively determines the rate of granulosa cell apoptosis [28]–[30]. Therefore, immunoreactive BimEL staining in granulosa cells was performed to evaluate whether the BimEL expression is related to the serum testosterone level in endometriosis patients. Typical examples are shown in Fig. 1. The FSH immunoreactivity was shown in granulosa cells of growing follicles (Fig. 1A–c, –f, Fig. 1B–c, and –f). BimEL staining was shown in granulosa cells in Fig. 1B–d and –g, however, absence of BimEL expression was observed in Fig. 1A–d and –g. The specimens with a BimEL-positive rate higher than 20% in the follicles were classified as the BimEL-positive group (n = 6) and the specimens with staining of less than 20% of the specimen were classified as the BimEL-negative group (n = 4). As shown in Table 2, there were no significant differences among the BimEL-positive and negative groups in age, or in the serum FSH, LH or estradiol concentrations, the size of endometrioma or the serum CA125 level at surgery. However, the mean serum testosterone level was 0.33±0.26 ng/ml in the BimEL-negative group, compared to the significantly lower value (0.15±0.21 ng/ml) observed in the BimEL-positive group.

**Table 2:** A comparison of the characteristics of the groups according to the BimEL staining status of primordial and growing follicles obtained from ovarian specimens in endometriosis patients.

	BimEL negative (n = 4)	BimEL positive (n = 6)	*p* value
Age (year)	35.1±1.8	36.2±3.6	NS
Serum FSH (mlU/ml)	9.8±21.2	10.4±15.7	NS
Serum LH (mlU/ml)	8.7±10.	5.0±12.5	NS
Serum Estradiol (pg/ml)	121.8±115.2	72.0±95.2	NS
Serum Testosterone (ng/ml)	0.33±0.26	0.15±0.21	0.008
Size of endometrioma (mm)	62.8±12.4	60.0±11.4	NS
Serum CA125 (U/mL)	92.9±52.4	90.0±46.4	NS

NS, not significant.

### Testosterone inhibited the apoptosis induced by testosterone depletion in the COV434 cells

**Figure 2:**
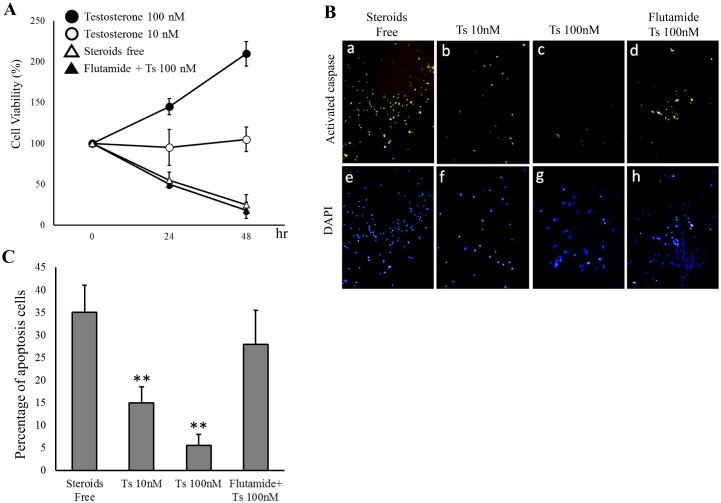
Testosterone inhibited the apoptosis induced by testosterone depletion in the COV434 granulosa cells. COV434 cells were incubated in phenol red-free DMEM supplemented with 2% Charcoal Stripped Fetal Bovine Serum (steroid-free conditions) for 12 hours. A: The cells were then incubated for 24 and 48 hours in the presence or absence of 10 or 100 nM testosterone with or without 5 µM of flutamide, and the cell viability was assessed using an MTS assay. B: The level of apoptosis of granulosa cells under various conditions was determined according to an activated caspase-3 assay using the CspACETM FITC-VAD-FMK In Situ Marker. FITC-labeled cells indicate the presence of apoptosis, and DAPI was used for counterstaining. C: A flow cytometry analysis was performed to count the number of FITC-labeled apoptotic cells using FACScan. Approximately 10,000-gated events were analyzed per sample. The data are representative of three independent experiments. Significant differences are indicated by an asterisk **p<0.01 compared to the Steroids Free, and Flutamide + Ts 100 nM.

In order to investigate the effects of testosterone on the granulosa cells, the cell viability was examined according to an MTS assay using the immortalized human granulosa cell line, COV434 cells. As shown in Fig. 2A, the viability of the COV434 cells was decreased under the steroid-free conditions (Fig. 2A, open triangle), whereas testosterone improved the cell viability in a dose-dependent manner (Fig. 2A, open and closed circles). An activated caspase-3 *in situ* detection assay was then performed to evaluate whether testosterone depletion reduces the cell viability by inducing the apoptosis of COV434 cells. While testosterone depletion increased the number of FITC-labeled apoptotic cells (Fig. 2B–a), testosterone administration decreased the number of apoptotic cells (Fig. 2B–b and B–c). In addition, treatment with flutamide, an androgen receptor antagonist, attenuated the anti-apoptotic effect of testosterone (Fig. 2B–d). In order to quantify the level of apoptosis induced by testosterone depletion, approximately 10,000-gated events were analyzed using flow cytometry. The rate of apoptosis among the COV434 cells incubated under steroid-free conditions was 34.8±6.3%; however, the administration of testosterone significantly decreased this value to 15.5±3.4% for treatment with 10 nM testosterone and 6.2±2.9% for treatment with 100 nM testosterone (Fig. 2C). The administration of flutamide was subsequently used to confirm the testosterone-specific effect on the inhibition of apoptosis. Consequently, co-treatment with 100 nM testosterone and 5 µM of flutamide failed to improve the cell viability (Fig. 2A, closed triangle) and/or inhibition of apoptosis (Fig. 2B–d and 2C) induced by testosterone depletion in the COV434 cells.

### Testosterone inhibited the apoptosis induced by testosterone depletion via the PI3K/Akt-FoxO3 cascade in the COV434 cells

**Figure 3:**
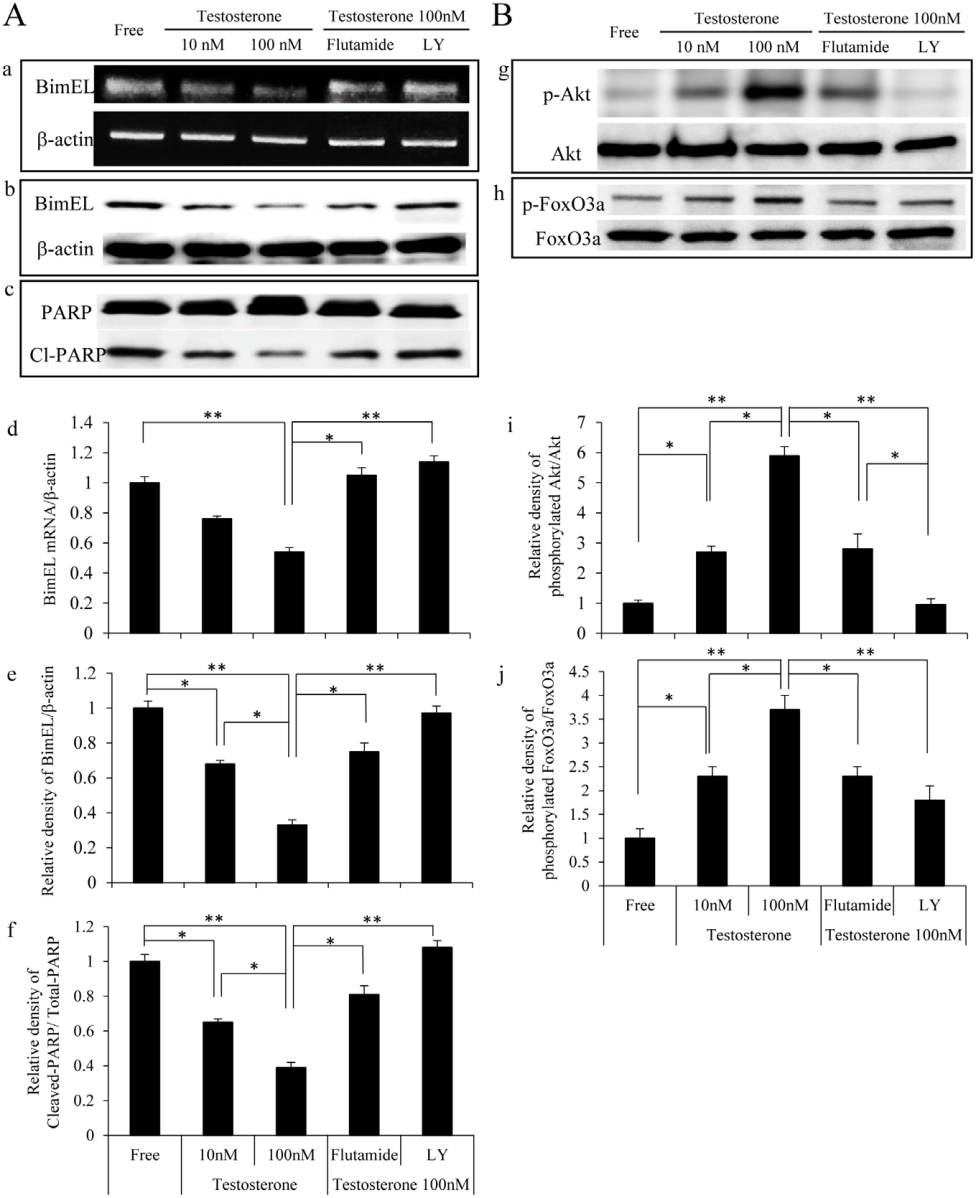
Testosterone inhibited the apoptosis via the Akt-FoxO3a pathway in the COV434 granulosa cells. COV434 cells were incubated in phenol red-free DMEM supplemented with 2% Charcoal Stripped Fetal Bovine Serum (steroid-free conditions; Free) for 12 hours. The COV434 cells were then treated with vehicle or 10 or 100 nM testosterone for 16 hours for the RT-PCR analysis and 24 hours for Western blotting in the presence or absence of flutamide (10 µM) or LY294002 (25 µM) for one hour. A: (a) The mRNA expression of BimEL was determined using RT-PCR, and the beta-actin mRNA expression was used as an internal loading standard. Relative densitometric units of BimEL to beta-actin are shown in panel (d). The cell lysates were analyzed according to Western blotting using antibodies to BimEL (b), PARP (c) or beta-actin. Relative densitometric units of BimEL to beta-actin, and cleaved-PARP to total PARP are shown in panel (e) and (f), respectively. B: The cell lysates were also analyzed according to Western blotting using antibodies to phosphorylated-Akt, Akt (g), phosphorylated-FoxO3a or FoxO3a (h). Relative densitometric units of phosphorylated-Akt to total Akt, and phosphorylated-FoxO3a to total FoxO3a are shown in panel (i) and (j), respectively. Values shown represent the mean ± s.e. from at least three separate experiments. Significant differences are indicated by asterisks. *, p<0.05, **, P<0.01.

In order to investigate the role of testosterone in inhibiting the apoptosis induced by testosterone depletion in the COV434 cells, the expression levels of the pro-apoptotic factors BimEL and cleaved-PARP were evaluated, as shown in Fig. 3A. Both the mRNA and protein levels of BimEL increased under the testosterone-free conditions; however, the levels of these markers subsequently decreased following the addition of testosterone in a dose-dependent manner (Fig. 3A, upper and middle panels). Similarly, although the expression of cleaved-PARP was detected under the testosterone-free conditions, testosterone treatment reduced the levels of this marker in a dose-dependent manner (Fig. 3A, lower panel).

Recent reports have demonstrated that the expression of the pro-apoptotic marker BimEL is regulated by a forkhead transcriptional factor, forkhead box O3 (FoxO3a) [31], [32]. The unphosphorylated form of FoxO3a activates BimEL transcription via a FoxO-binding site, whereas activation of the PI3K/Akt pathway phosphorylates FoxO3a following the inhibition of its transcriptional activity. Therefore, we examined whether testosterone phosphorylates FoxO3a via the PI3K/Akt pathway. Although treatment with testosterone did not affect the expression of Akt or FoxO3 proteins, both Akt and FoxO3 were rapidly phosphorylated by testosterone in a dose-dependent manner in the COV434 cells (Fig. 3B). Meanwhile, the testosterone–induced Akt and FoxO3 phosphorylation was attenuated by both flutamide and LY294002, suggesting that testosterone induces Akt-FoxO3 phosphorylation via AR and PI3K. Furthermore, the reduction in the BimEL and cleaved-PARP expression levels induced by testosterone was also attenuated by flutamide and LY294002. Taken together, the administration of testosterone inhibits the apoptosis induced by testosterone depletion via PI3K/Akt-FoxO3 activation following inhibition of the BimEL expression.

## Discussion

Several mechanisms have been proposed for the association between endometriosis and infertility. Suspected contributing factors to reduced fertility include an altered peritoneal function resulting in impaired folliculogenesis and oocyte quality, pelvic anatomy distortion, immunologic dysfunction and impaired implantation [33]. The impact of endometriosis on the quality of oocytes has been assessed in studies evaluating the characteristics of donor oocytes of patients with and without endometriosis and the implantation rates in recipients. Although previous reports have demonstrated low testosterone levels in the follicle fluid in patients with endometriosis [18] and in the serum in patients with POI [34], the mechanisms underlying the decrease in the testosterone levels in such patients have not been elucidated.

In the current study, we showed that patients with endometriosis have significantly lower serum testosterone levels than those without endometriosis. Several reports have demonstrated that the serum testosterone levels are not decreased in patients with endometriosis [18], [35]. However, there were differences in the age of the subjects, the time of blood sampling and the study populations in these previous study. In the present study, we examined subjects who were relatively old, at 35 years old on average, who might be in Stage-3, corresponding to late reproductive phase, based on the STRAW (Stage of Reproductive Aging Workshop) criteria [36], [37]. Therefore, the impact of endometriosis on the ovarian reserve in our study might be larger compared to studies considering younger populations.

Our study has demonstrated that high positive rates for pro-apoptotic BimEL staining in the follicles are significantly correlated with a lower serum testosterone level in endometriosis patients (Table 2). Furthermore, our *in vitro* examinations showed that testosterone depletion induces the apoptosis of granulosa cells, whereas this process is inhibited by the administration of testosterone. In previous reports, testosterone has been shown to enhance follicular recruitment [38], promote follicular growth and development [39], increase the insulin-like growth factor 1 (IGF-1) expression in the primate ovary, which plays an essential role in regulating follicular development [40], and increase follicular sensitivity to FSH stimulation via androgen receptors [41], [42]. Our current data are in accordance with recently published evidence suggesting that testosterone is essential for normal follicular development.

In the current study, COV434 cells were used to clarify the mechanisms underlying the anti-apoptotic effects of testosterone. The primary culture of granulosa cells obtained from the patients undergoing *in vitro* fertilization (IVF) limited the number of cells obtained, and these could not be cultured longer than a few days. In addition, these cells are usually fully luteinized at the time of isolation due to hyperstimulation with gonadotropins during the assisted reproductive therapy. The aim of our study was clarify the mechanisms responsible for the protective effects of testosterone in the earlier stage of follicle maturation. Therefore, we decided to use an established cell line, COV434, in the current *in vitro* examinations. These experiments have demonstrated that COV434 cells exhibited physiological responses similar to primary human granulosa cells [23], [43]. In addition, we have investigated whether the properties of the COV434 cells are in agreement with the characteristics of granulosa cells. As shown in S1 Figs. A and B, COV434 expressed mRNA and protein for both the AR and FSHR. In addition, COV434 cells secreted 17β-estradiol in response to FSH stimulation under the condition of 20 ng/ml testosterone (S1 Fig. B). Therefore, the COV434 cells served as an excellent model in our study.

The actions of testosterone are mediated via the classical receptor, androgen receptor (AR), in the nucleus or on the membrane. Rapid non-genomic signaling of testosterone was observed in the current study, as Akt was phosphorylated for five minutes. Furthermore, the Akt phosphorylation induced by testosterone was strongly attenuated by pretreatment with the AR inhibitor flutamide, suggesting that non-genomic testosterone signaling is induced via AR. FoxO3, a member of the FOXO subfamily of forkhead transcription factors, is primarily expressed in the nuclei of oocytes in primordial follicles [44] and participates in diverse processes, including cell apoptosis [45]. The transcriptional activity of FoxO3 is inhibited by the phosphorylation of this factor via the PI3K-Akt pathway in leukemia cells [31], [46]. The pro-apoptotic protein Bim was recently identified and shown to promote the apoptosis of thymic lymphoma cells [47]. Interestingly, Wang et al. demonstrated that BimEL induces porcine granulosa cell apoptosis during follicular atresia and its expression is regulated via the PI3K/Akt/FoxO3a pathway [48]. In the present study, conditions of testosterone depletion enhanced the expression of BimEL, which was subsequently inhibited by the administration of testosterone. In addition, the inhibition of the BimEL expression following the administration of testosterone was blocked by both an androgen receptor inhibitor (flutamide) and LY294002. Furthermore, testosterone phosphorylated Akt and FoxO3a proteins, which was consequently blocked by flutamide and LY294002. Taken together, testosterone plays a role in regulating the apoptosis of granulosa cells via phosphorylation of the PI3K/Akt/FoxO3a pathway in a non-genomic manner following inhibition of the BimEL expression. Therefore, in the present report, we elucidated, for the first time, the mechanisms underlying the anti-apoptotic effects of testosterone on granulosa cells. The major question remaining is why there is a low testosterone status in endometriosis patients. There has been no evidence that endometriosis attenuates the function of the theca cells, which are the major source of androgens in the ovaries. To examine the pathology associated with the low testosterone induced by endometriosis in greater detail, measurement of androstenedione, which is a precursor of testosterone, in the serum and follicle fluid will be required. In addition, to evaluate the adrenal function, the measurement of dehydroepiandrosterone may also be necessary.

In summary, we herein demonstrated that patients with endometriosis have significantly lower serum testosterone levels than those without and that a low serum testosterone level may induce the apoptosis of granulosa cells in these patients. A recent meta-analysis by Bosdow et al. including two RCTs [49], [50] demonstrated that pretreatment with transdermal testosterone is associated with increased rates of clinical pregnancy and live birth and reduces the dose of FSH required in poor responders undergoing ovarian stimulation for IVF [51]. Although testosterone replacement therapy for endometriosis patients with low serum testosterone may help to prevent POI, further investigations are needed to confirm these findings.

## Supporting Information

S1 Fig **Characteristics of the COV434 cells.** A: The expression of the AR and FSHR in COV434 cells. (a) The mRNA expression of the AR and the FSHR was determined using RT-PCR, and the beta-actin mRNA expression was used as an internal loading standard. (b) The protein expression of the AR and FSHR was determined by a Western blot analysis. Cell culture media were collected after incubation with 200 ng/ml of FSH and 20 ng/ml of testosterone for 24 h and 48 h. The concentrations of estradiol and testosterone in the culture media were assayed by ELISA. Estradiol was generated and testosterone was reduced in the culture medium in the presence of 200 ng/ml FSH. This result indicates that testosterone in the culture medium was converted to estradiol by aromatase in the COV434 cells.
(TIF)

S1 Materials and Methods **Materials and methods.**
(DOC)
