# Supplementary figures and images for: A Low-Testosterone State Associated with Endometrioma Leads to the Apoptosis of Granulosa Cells
Source: PLoS One. 2014 Dec 23;9(12):e115618. doi: 10.1371/journal.pone.0115618 (PMC4275210; doi:10.1371/journal.pone.0115618)

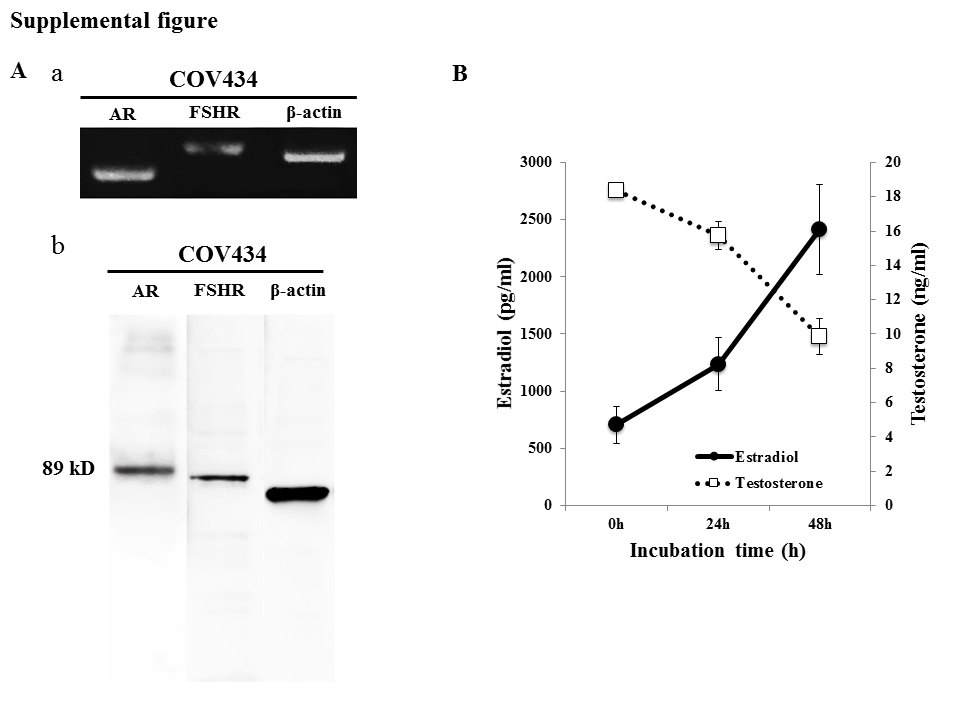

Supplement: S1 Fig — Characteristics of the COV434 cells. A: The expression of the AR and FSHR in COV434 cells. (a) The mRNA expression of the AR and the FSHR was determined using RT-PCR, and the beta-actin mRNA expression was used as an internal loading standard. (b) The protein expression of the AR and FSHR was determined by a Western blot analysis. Cell culture media were collected after incubation with 200 ng/ml of FSH and 20 ng/ml of testosterone for 24 h and 48 h. The concentrations of estradiol and testosterone in the culture media were assayed by ELISA. Estradiol was generated and testosterone was reduced in the culture medium in the presence of 200 ng/ml FSH. This result indicates that testosterone in the culture medium was converted to estradiol by aromatase in the COV434 cells. (TIF) [file pone.0115618.s001.tif]
