# Supplementary material for: A Low-Testosterone State Associated with Endometrioma Leads to the Apoptosis of Granulosa Cells
Source: PLoS One. 2014 Dec 23;9(12):e115618. doi: 10.1371/journal.pone.0115618 (PMC4275210; doi:10.1371/journal.pone.0115618)
Supplement: S1 Materials and Methods — Materials and methods. (DOC) [file pone.0115618.s002.doc]

**Supplemental Materials and Methods**

Materials

The rabbit monoclonal anti-human androgen receptor antibodies and rabbit monoclonal　anti-human FSH receptor antibodies used for immunoblotting were purchased from Abcam (Cambridge, MA, USA).

RNA extraction, cDNA synthesis and RT-PCR

Total RNA was obtained from cultured cells using the RNeasy Mini kit (Qiagen, Germantown, MD, USA), and 2 μg was subsequently reverse-transcribed with Superscript II RNase H-reverse transcriptase (Invitrogen, Carlsbad, CA, USA) using random primers, according to the manufacturer’s instructions. cDNA was then amplified using Taq DNA polymerase (Roche Diagnostics, Mannheim, Germany). The primer sequenced used were as follows: AR: 5’-TGTCGTCTTCGGAAATGTTATGA -3’ (forward) and 5’-TTCCTCCTGTAGTTTCAGATTACCA-3’ (reverse), FSHR: 5’-TGCCATTCAATGGAACCCAACT -3’ (forward) and 5’-CGTGGAAAACATTAGGCAAT-3’ (reverse), Beta-actin: 5’-AGC CAC ATC GCT CAG ACA-3’ (forward) and 5’-GCC CAA TAC AC CAA ATC C-3’ (reverse).

Western blot analysis

COV434 cells were incubated in phenol red-free DMEM supplemented with 2% charcoal-stripped fetal bovine serum for 12 hours. The cells were subsequently washed twice with ice-cold phosphate-buffered saline and lysed using Pierce RIPA Buffer (Thermo Fisher Scientific, MA, USA). Equal amounts of whole cell proteins were separated via SDS polyacrylamide gel electrophoresis and electrotransferred to nitrocellulose membranes. The Western blot analyses were then performed with various specific primary antibodies; the immunoreactive bands in the immunoblots were visualized with horseradish peroxidase-coupled immunoglobulin using an enhanced chemiluminescence Western blotting system (ECL Plus, GE Healthcare Life Sciences, Pittsburgh, PA, USA).

Enzyme-linked immunosorbent assay (ELISA)

COV434 cells were seeded at 1×10^6^ cells per well in six-well plates and were cultured with growth media until they reached 70% to 80% confluence. The cells were then starved for 16 hours. Culture supernatants were collected after being incubated for 24h or 48 h with 200 ng/ml FSH and 20 ng/ml of testosterone. Estradiol and testosterone were assayed with 100 μl of cell free culture supernatant using an estradiol ELISA kit and a testosterone ELISA kit (American Laboratory Products Company, Salem, NH) according to the manufacturer’s instructions. The absorbance was read at 450 nm with the Corona SH-1000 lab absorbance microplate reader (Corona Electric Co.Inc, Ibaraki, Japan). Sample concentrations were determined by interpolation from the standard curve. The assay was performed three times, and the ratio was expressed as the mean ± SD.
